# Conditioning Strategies for Improving Handball Throwing Velocity: A Systematic Review and Meta-Analyses

**DOI:** 10.5114/jhk/162017

**Published:** 2023-04-20

**Authors:** Jan Petruzela, Monika Papla, Petr Stastny

**Affiliations:** 1 Faculty of Physical Education and Sport, Charles University, Prague, Czech Republic.; 2 Institute of Sport Sciences, The Jerzy Kukuczka Academy of Physical Education in Katowice, Katowice, Poland.

**Keywords:** core training, resistance training, team sports games, ball velocity

## Abstract

Ball throwing velocity is essential for scoring goals in handball; the crucial question is how to develop throwing velocity in highly trained handball players. Therefore, this systematic review aims to summarize effective conditioning strategies to improve throwing velocity in elite male players and to perform a meta-analysis on which training system can provide the highest increase in throwing velocity. The literature was analyzed using the Preferred Reporting Items for Systematic Reviews and Meta-analyses (PRISMA) in PubMed, Scopus, and Web of Science. Thirteen studies (sample n = 174) were included: five resistance training studies, one core training study, one study on repeated shuffle sprint training with small-sided games, and one on eccentric overload training. Effect size comparison showed that resistance training is the most effective strategy for improving throwing velocity in elite handball players (d > 0.7). Core training showed a small effect (d = 0.35). Small-sided game (SSG) training showed different results, from a significant positive effect (d = 1.95) to a negative effect (d = –2.03), and eccentric overload training showed a negative effect (d = –0.15). Resistance training is the most effective strategy for improving throwing velocity in elite handball players, while core training and SSGs can improve throwing velocity in youth athletes. Due to the small number of studies focusing on elite handball players, there is a need for more studies on advanced resistance training methods, e.g., contrast, complex, ballistic training, because much greater demands are placed on handball performance assumptions.

## Introduction

Team handball is a sport in which players must repeatedly produce actions with maximal or submaximal efforts without full recovery between these actions. The most common handball actions are sprinting, jumping, and throwing (Rios et al., 2021), where ball throwing is considered a fundamental element (Zapartidis et al., 2007) essential for scoring goals (Rios et al., 2021), and should not differ between game periods (Zapardiel Cortés et al., 2017). Generally, a faster throw provides less time for goalkeepers and defenders to react. There are many strategies used in handball practice to increase throwing velocity, and strength, speed, and power training have been evaluated for their effectiveness; however, a summary of different training procedures has not yet been performed.

Previous reviews (Bragazzi et al., 2020; Vila and Ferragut, 2019) have attempted to summarize the effects of conditioning on throwing velocity without considering players' performance. A greater stature, mass, elbow angles, along with higher elbow displacement during ball release, hand size, biacromial breadth, and finger length have been associated with higher throwing speed (Aguilar-Martínez et al., 2012; Vila and Ferragut, 2019). It was also found that resistance training increased throwing velocity without losing accuracy (Bragazzi et al., 2020). However, the selection of training methods remains to be determined due to heterogeneous study groups in original studies and inconsistent methodological approaches. For example, one study did not find the positive effects of resistance training on elite athletes (Sabido et al., 2017), while another study on non-elite athletes did find such positive effects (Kotzamanidis, 2003; Vila and Ferragut, 2019). Generally, different training interventions including resistance training, core training or circuit training improve throwing speed (Hermassi et al., 2010, 2011; Vila and Ferragut, 2019), but there is no clear recommendation on which intervention is the most effective or recommended for elite handball players. Since the handball throw is realized at high speed and acceleration, power and resistance training should be effective for the development of throwing speed (Behm et al., 2017)

To improve power in strength training programs, there is always a conflict between using heavier or lighter loads (Abuajwa et al., 2022), with the principal question of whether athletes have a sufficient level of maximal strength to benefit from explosive training. In general, it is recommended that strength development should be prioritized over explosiveness in adolescent athletes (Behm et al., 2017). It has been reported that resistance training >80% of 1RM has a positive effect on throwing speed in adolescents (Gorostiaga et al., 1999), probably due to the close relationship between upper body strength and throwing performance (Van den Tillaar, 2004). On the other hand, another study suggests that lighter loads (intensity of 45% of 1RM) are as effective as heavy load training in improving strength (Lyttle et al., 1996). Therefore, the effects of different training methods and their intensity should be summarized for specific movement patterns, such as handball throws.

The development of throwing velocity must be based on a stable throwing technique and progressive development of strength, power, and speed (Cherif et al., 2016; Marques et al., 2007), which should be in line with fine motor control (Wagner et al., 2011). Therefore, various methods, such as heavy ball throws, light ball throws, plyometrics, and complex contrast training, are applied in sports practice (Escamilla et al., 2011; Szymanski, 2012). Each method can improve throwing velocity, especially for an athlete who does not reach a performance plateau. However, there is still the question of how to develop throwing velocity in highly trained male handball players. Therefore, this systematic review aimed to summarize the effective conditioning strategies to improve throwing velocity in elite players, and to perform a meta-analysis on which training system can provide the greatest increase in throwing velocity. We hypothesized that the highest effect would be from resistance training methods with moderate and high intensities (over 45%) of 1RM.

## Methods

### Literature Search

A literature search was conducted according to PRISMA in Scopus, PubMed, and Web of Science on January 6^th^, 2021. The search strategy included the words “handball”, “measurement”, “exercise”, and “test”, and all variants of the words were used with the same base. The complete strategy with Boolean operators was “Handball* and (exercise* or test* or measurement* or throwing*)”. There were no language or date limits.

### Inclusion Criteria

The PECOS (Participants, Exposition, Comparators, Outcomes, Study design) inclusion criteria were as follows: 1) the use of elite men's handball players as participants (playing in the first league), 2) participants were exposed to conditioning intervention, 3) studies compared throwing velocity before and after the intervention, and 4) the outcome was expressed in raw values of throwing velocity or throwing velocity difference. Criterion 1 (elite status) was determined based on the statement in the original study, whether players were top national level league players, which meant that they played in the highest domestic competition.

The inclusion criteria were used during the title and abstract screening, and the following exclusion criteria were used during full-text screening: 1) handball players under 18 years old and players who were not top-level athletes (based on reported division and region), 2) participants were not exposed to the intervention, 3) participants were not tested on throwing velocity before and after the intervention, and 4) the essential outcomes of throwing velocity were missing (standard deviation data, pre/post data), 5) the study referred to high throwing testing reliability (ICC > 0.8).

We chose studies where throwing speed was expressed in m/s or km/h before and after the intervention, and every expression was required to have an associated standard deviation value. Both criteria had to be reported in at least one of these three types of throws: a 3-step run throw, a jump throw, and a standing throw. The PEDRO scale checked the general quality of the included studies.

### Statistical Analysis

An Excel sheet was used to calculate the statistical data. The mean and standard deviation of pre- and post-intervention values were exported to an Excel file, where Cohen’s *d* (Cohen, 1988) was used to determine the throwing difference effect size (ES). The scale estimated an ES of less than 0.41 as a small effect, 0.41–0.70 as a moderate ES, and more than 0.70 as a significant ES (Cohen, 1988). The ES, 95% confidence interval, standard deviation and standardized mean difference (SMD) were calculated from the mean before and after the training intervention.

## Results

**Figure 1:**
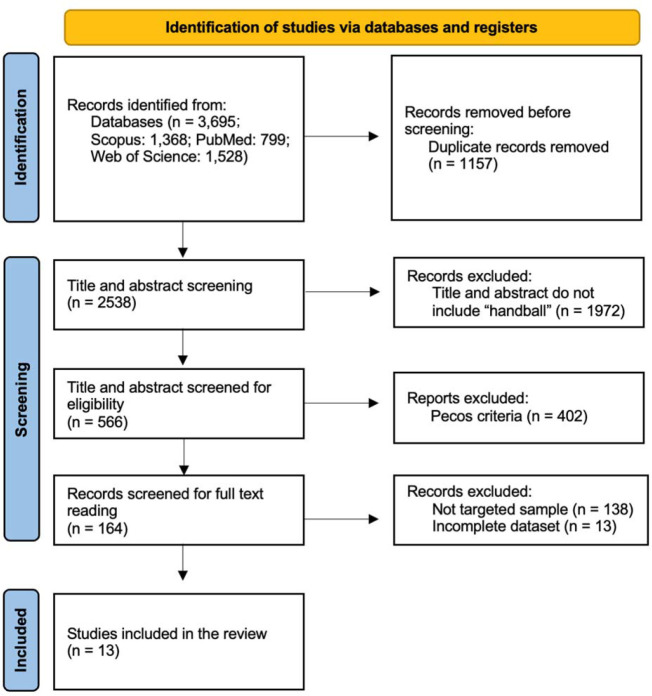
The flow chart of literature search and eligibility screening of studies reporting the effects of training intervention on handball throwing speed.

The total number of searched studies was 3 695 (Scopus: 1 368 studies, PubMed: 799 studies, Web of Science: 1 528 studies). After duplicates were removed, 2 538 studies remained (Figure 1).

A total of 1972 studies in which “handball” was not included in the title or the abstract were removed. Of the remaining 566 studies, 402 studies that did not meet the requirements of the PECOS criteria of a systematic review were removed. Of the remaining 164 studies, 151 studies were excluded during the full-text reading. Reasons for exclusion were gender, age, players’ performance level, and missing data (standard deviation, mean, pre/post data). Thirteen studies (Hermassi et al., 2010, 2011, 2015, 2019a, 2019b, 2019c; Cherif et al., 2016; Iacono et al., 2016; Manchado et al., 2017; Marques and González-Badillo, 2006; Sabido et al., 2017; Spieszny and Zubik, 2018) were included in the systematic review, and eight studies were included in the meta-analysis (Table 1). We did not find any studies that met the requirements of the meta-analysis via hand search.

**Table 1:** The basic characteristics of the included studies reporting the effects of the training modality on handball throwing speed.

Authors, Year	Participants (n, age, height, body mass)	Player’s performance level	Intervention	Main aims	Conclusion
Marques and Gonzalez-Badillo, 2006	n = 16 age: 23.1 ± 4.7 height: 184.2 ± 13.1 weight: 84.8 ± 13.1	Elite team handball players	RT for 12 weeks (2–3 x per week): Parallel squat, Countermovement jump onto a box, Bench press, sprints	To investigate changes in physical variables produced during in-season resistance training and detraining in 16 high-level team handball players	Elite team handball players can optimize important physical variables over 12 weeks of in–season training
Hermassi et al., 2010	n = 26 age: 20.0 ± 0.6 height: 186.0 ± 6 weight: 85.0 ± 13.2	Players from the top national handball league	Heavy and moderate RT for 10 weeks (2 x per week): Bench press, pullover	To compare the effect of 2 differing 10-week resistance training programs on peak power output, muscle volume, strength, and throwing velocity of the upper limbs in handball players during the competitive season	During the competitive season, peak power, maximal strength, and throwing velocity of male handball players were increased more by 10 weeks of bench press and pullover training with suitably adapted heavy loads than with moderate loads
Hermassi et al., 2011	n = 24 age: 22.1 ± 1.7 height: 182.0 ± 7 weight: 81.1 ± 14.7	Elite national level handball players	RT 8 for weeks (2 x per week): Bench press, pullover, half back squat	To test the potential of in-season heavy upper and lower limb strength training to enhance peak power output, vertical jump, and handball-related field performance in elite male handball players who were already well-trained, and to assess any adverse effects on sprint velocity	Biweekly heavy back half-squat, pullover, and bench-press exercises can be recommended to elite male handball players as improving many measures of handball-related performance without adverse effects upon the speed of movement
Manchado et al., 2017	n = 30 age: 18.7 ± 3.4 height: 179.3 ± 7 weight: 78.9 ± 7.7	Elite national level handball players	Core training (4 sessions per week): Crunch or curl-up with a Swiss ball, Cross curl up, Frontal bridge with a Swiss ball	To analyze the effect of a core training program on throwing velocity in 30 handball players	An increase in the strength and stability of the lumbopelvic region can contribute to the improvement in the kinetic chain of the specific movement of throwing in handball, thus, increasing throwing velocity
Hermassi et al., 2019a	n = 20 age: 20.9 ± 0.7 height: 184.0 ± 3 weight: 85.2 ± 8.8	First National League players	RT for 10 weeks (2 x per week): Clean and jerk, bench press, snatch, half-squat	To assess the effects of short-term resistance training and two weeks of tapering on physical performance in handball players.	Ten weeks of progressive resistance training followed by two weeks of tapering were an effective overall tactic to increase muscle power, sprint performance, and ball throwing velocity in handball players
Hermassi et al., 2019b	n = 22 age: 20.3 ± 0.5 height: 183.0 ± 7 weight: 84.8 ± 7.6	Players from top national handball league	Resistance circuit training for 10 weeks (2 x per week): Frontal jumps over barrier, Bench press, half squat, fontal sprint, hurdle jump, pullover, zig-zag sprint, depth jumps	To analyze the effects of a resistance-type circuit training program on male handball players	During the competitive season, 10 weeks of resistance circuit training with only two training sessions per week improved numerous measures of athletic performance in handball players; such conditioning can be highly recommended as part of the annual training program for elite handball players
Iacono et al., 2016	n = 18 age: 24.8 ± 4.4 height: 183.0 ± 7 weight: 84.8 ± 7.6	Elite team handball players	Small-sided games and repeated shuffle sprint training for 8 weeks (twice per week)	To compare the effects of small-sided games and repeated shuffle sprint training on repeated sprint ability and countermovement jump test performances of elite handball players	Small-sided games seem to be more effective in improving agility and standing throws, whereas repeated shuffle sprint training seems preferable in improving 10-m sprint, CMJ, and jump shot performance
Sabido et al., 2017	n = 18 age: 23.9±3.8 height: 183.0 ± 7 weight: 79.5±7.7	Players from top national handball league	Eccentric-overload training for 7 weeks (once per week): bilateral half squats, lunges	To investigate the influence of adding a weekly eccentric-overload training session in several athletic performance tests	Groups did not show changes in handball throwing velocity

RT = resistance training

After excluding studies that did not match the criteria, thirteen remained. Four studies investigated the effects of resistance training, one study investigated the effects of circuit training, one study investigated the effects of repeated shuffle sprints and small-sided games, one study investigated the effects of core training, and one study investigated the effects of eccentric overload training.

### Meta-Analysis Results

**Figure 2:**
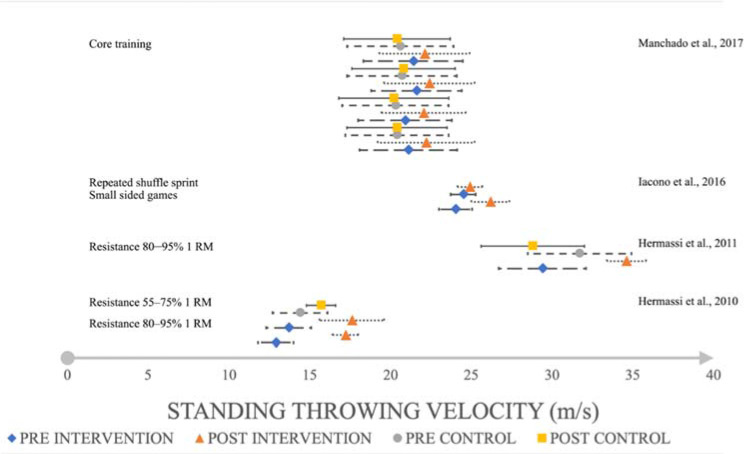
The impact of training interventions on standing throwing velocity (m/s).

**Figure 3:**
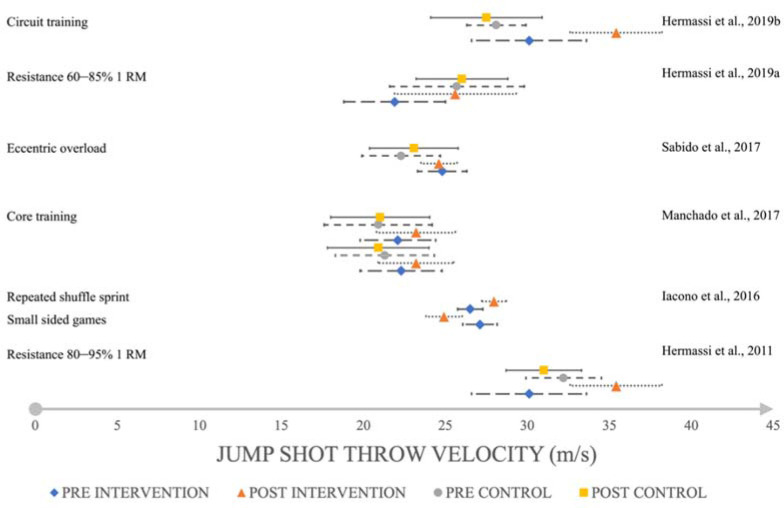
The impact of training interventions on jump shot velocity (m/s).

**Figure 4:**
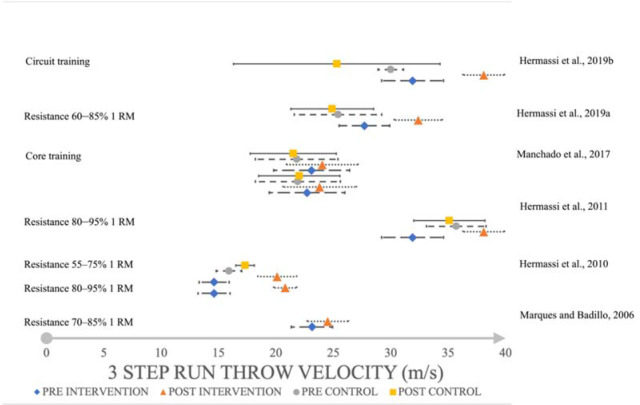
The impact of training interventions on 3-step run throwing velocity (m/s).

Resistance training increased the throwing speed with a large effect (*d* = 4.47,SMD = 4.36, 95%CI 16.677–17.723; *d* = 2.25, SMD = 2.19, 95%CI 16.293–18.907; *d* = 2.48, SMD = 2.21, 95%CI 33.921–35.279; Figure 2) in the standing throw, a large effect in the jump shot (*d* = 1.67, SMD = 1.65, 95%CI 33.816–36.984; *d* = 1.08, SMD = 1.07, 95%CI 23.307–27.893, Figure 3) and a large to very large effect in the 3-step run throw (*d* = 0.76, SMD = 0.76, 95%CI 23.618–25.382; *d* = 5.09, SMD = 4.96, 95%CI 20.147–21.453; *d* = 3.63, SMD = 3.57, 95%CI 28.994–31.206; *d* = 2.70, SMD = 2.6, 95%CL 37.082–39.118; *d* = 2.18, SMD = 2.18, 95%CI 31.098–33.702; *d* = 2.70, SMD = 2.60, 95%CI 37.082–39.118; Figure 4). On the other hand, eccentric overload training harmed jump shot throwing speed at a very small effect size (*d* = –0.15, SMD = –0.14, 95%CI 23.950–25.250; Figure 2).

Core training increased throwing speed with a small effect (*d* = 0.36, SMD = 0.29, 95%CI 20.682–23.718; *d* = 0.41, SMD = 0.42, 95%CI 20.734–23.366; *d* = 0.28, SMD = 0.29, 95%CI 20.983–23.817; *d* = 0.23, SMD = 0.24, 95%CL 20.683–23.517; Figure 2) in the standing throw, a small effect (*d* = 0.33, SMD = 0.31, 95%CI 22.181–25.419; *d* = 0.28, SMD = 0.27, 95%CI 22.446–25.594; Figure 4) in the 3-step run and a medium to small effect (*d* = 0.37, SMD = 0.37, 95%CI 22.036–24.364; *d* = 0.46, SMD = 0.47, 95%CI 21.985–24.415; Figure 3) in the jump shot.

**Figure 5:**
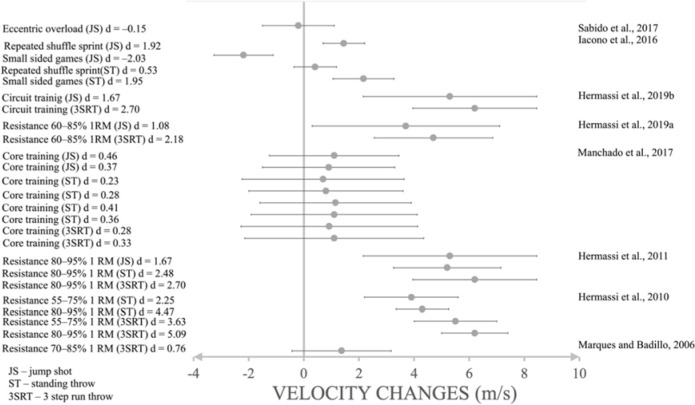
The throwing velocity changes after different types of training interventions (m/s).

Small-sided games had a large effect (*d* = 1.95, SMD = 1.94, 95%CI 25.409–26.951; Figure 2) on increasing standing throwing velocity, but a negative, very large effect (*d* = –2.03, SMD = –2.01, 95%CI 24.191–25.629; Figure 3) on the jump shot. Repeated shuffle sprint training increased throwing speed with a medium effect (*d* = 0.53, SMD = 0.53, 95%CI 24.407–25.413, Figure 2) on standing throws, and a very large effect (*d* = 1.92, SMD = 1.92, 95%CI 27.470–28.450; Figure 3) on the jump shot. The most noticeable effect on mean throwing velocity on every type of shoot was due to resistance training and circuit training (Figure 5). Eccentric overload training and small-sided game training showed a negative effect on mean throwing velocity changes in jump shots (–2.19 m/s; –0.2 m/s; Figure 5) (Hermassi et al., 2010, 2011, 2019b, 2019c; Iacono et al., 2016; Manchado et al., 2017; Marques and González-Badillo, 2006; Sabido et al., 2017).

## Discussion

The main aim of this paper was to determine the most effective conditioning strategies to improve throwing velocity in elite players, and to perform a meta-analysis to determine which training program can provide the most significant increase in throwing velocity. Based on rigorous criteria, our results showed that resistance training interventions with moderate and high intensity (>55% of 1RM) were the best strategy to improve throwing velocity. Therefore, we can confirm our hypotheses, even though we did not find a study reporting the intensity between 45 and 55% of 1RM. Our results agree with a study on male adult collegiate handball players (not elite) who benefited in throwing velocity with intensity of 40% of 1 RM and 60% of 1RM (Abuajwa et al., 2022). Although moderate resistance training intensity might be sufficient, experienced players should especially benefit from higher intensities (intensity of >80% of 1RM) based on the principle of recruiting high-threshold fast-twitch motor units (McBride et al., 2002). In contrast, core training had a minimal effect on throwing velocity (Manchado et al., 2017) (Figure 5). The results support that exercise intensity can be a major factor in the group of elite handball players, supporting the results from other sports, e.g., baseball and football (Szymanski, 2012).

Our results underline a close relationship between speed and strength in specific movement patterns in trained individuals (Koźlenia et al., 2020), and might be explained by high threshold neural drive stimulation created by resistance training. Our findings also follow the recommendations from a previous meta-analysis (Behm et al., 2017), which suggests that power training should be preceded by maximal strength development. Considering these facts and results, we emphasize that strength training developed in youth categories is crucial in developing power in later handball training phases. However, other methods, such as sprint intervals or circuit training, can also improve throwing speed, supporting or preceding resistance training. From the point of view of throwing velocity development, we could not find studies with training focusing on intensity contrast (such as postactivation performance enhancement, PAPE), complex and ballistic methods, or specific resistance training methods using, e.g., dumbbells and the Keiser cable system, which have been suggested to be effective in non-elite baseball, soccer, and handball (Escamilla et al., 2011; Markovic et al., 2008; Szymanski, 2012). Using PAPE protocols in handball players was an effective strategy for lower limb power development (Dello Iacono et al., 2018). Therefore, it should have a positive effect on throwing velocity as well. However, future research should investigate PAPE effectiveness concerning the strength level of handball athletes (Bellar et al., 2012).

The core training method showed a small positive effect, though it does not include strengthening a primary mover for handball throwing. In early training phases in youth categories, players can benefit from core training, but its efficiency is primarily for muscle stabilization (Jebavy et al., 2020) and lasts for a few weeks(Jebavý, 2012). Core training’s intensity can be increased by progressive exercise selection and programming with progressive overloads in exercise intensity, including external loads. In support of this, one study using core training in female youth categories showed a small positive effect (improving less than 1 m/s on average in 6 weeks) on throwing velocity (Saeterbakken et al., 2011). One of our findings is that resistance training is more effective than core training; unfortunately, we would need more studies on elite handball players to confirm or disprove this conclusion. Core training has different benefits, such as improving torso stability, strength, and injury prevention (Handzel, 2003). Therefore, core training can be beneficial, especially for youth athletes. For elite athletes, it can serve as a supplement to traditional resistance training.

Eccentric overload training focusing on lower limbs is an effective strategy for improving lower limb power and strength; however, this training has no effects on the upper limbs (Sabido et al., 2017). Lower limb strengthening may affect throwing speed in youth athletes, but it does not have the same effect on elite handball players. Eccentric overload training on the upper limbs has been performed with no significant improvements in performance (Asencio et al., 2020). The final execution in handball (throw) occurs in the concentric phase of contraction. Focusing on a different type of contraction may be the reason for the insufficient functionality of PAPE protocols. The positive PAPE effect has been shown in studies focusing on the concentric phase of contraction (Bellar et al., 2012; Dello Iacono et al., 2016, 2018).

Running-based interventions had a similar effect on all throwing types except for repeated shuffle sprint training and small-sided game training. Repeated shuffle sprint training had a large positive effect on the jump shot (d = 1.92), and a medium effect on the standing throw (d = 0.52). Sprinting is the fastest demonstration of strength, where greater force production leads to greater jump performance, and affects subsequent ball throws (Saavedra et al., 2019). In contrast, small-sided game training showed a large negative effect on jump shots (d = –2.03), and a large positive effect on the standing throw (d = 1.95). Small-sided games are typically used for conditioning, changing and erratic situations, whereas jump shots should not be used very often during this type of training. In contrast, change of direction is often used during small-sided game conditions, where players must absorb the forces several times higher than their body weight. Sometimes players have to stop in place, and building the capacity in this manner could be the main reason why SSG training positively affects standing throws. Therefore, SSG training is useful for building general and specific fitness (Laursen and Buchheit, 2019), when applied twice a week for eight weeks (Iacono et al., 2016). Another issue is that athletes' fitness levels affect throwing velocity, because they influence fatigue. One study investigated the influence of fatigue on throwing speed between the first and second halves in general, and there was no standard recovery time to guarantee that fatigue did not influence throwing speed (Zapardiel Cortés et al., 2017). Therefore, including a test that would consider fatigue during throwing velocity tests (Iacono et al., 2016) could be useful.

Some limitations of this meta-analysis include focusing on elite handball players, which are reported in relatively small quantities and sample sizes. One study had to be excluded due to missing standard deviation data (Hermassi et al., 2015).

The results showed improvement in throwing velocity via core training and small-sided games, which might mean that non-elite handball players were used in some studies despite reporting elite status or unclear definitions of top handball players.

If we look closer at the samples, we cannot find players from top world leagues, e.g., players from Germany, France, Spain, or Scandinavia. Each of these countries has a sophisticated system for developing strength at all ages. The results from the study of the Denmark national team in 2008 support that strength training and conditioning have a strong history there (Kvorning et al., 2017). Some interesting facts about performance were shown in the results of reported bench press 1RM (BP 1RM) in Hermassi studies from 2011–2019 and the Denmark's national team. Data from Hermassi et al.’s show (post results) 1RM BP 86.0 ± 9.9 kg (control group 78.1 ± 9.3 kg), 82.3 ± 7.5 kg (control group 77.6 ± 9 kg), 92.5 ± 6.2 kg (control group 68.0 ± 5.1), 80.4 ± 5 kg (control group 79.4 ± 5.4 kg (Hermassi et al., 2011, 2019b, 2019c, 2019d). We found that athletes in the Denmark's national team had greater maximal strength in the 1 RM bench press (BP 1RM = 110 ± 12.1 kg) compared to every other handball study. Considering that the bench press showed a medium correlation (r = 0.637; r = 0.55) with throwing velocity, we can indicate that superior strength of Danish athletes and not quite the elite level of players who are described in studies as top-level athletes (Debanne and Laffaye, 2011; Marques et al., 2007). As previously mentioned (Bragazzi et al., 2020), high-quality investigations are highly needed in handball world.

## Conclusions

Training for developing handball throwing velocity should consist of resistance exercises such as the bench press with an intensity higher than 55% of 1RM. Other exercises, such as a pullover and Olympic lifting, should be investigated for their relationship to handball throwing speed.

For elite players, details matter greatly, and it is not easy to increase throwing velocity. The previously mentioned methods (contrast, complex, ballistic, specific resistance training) should be tested for their effectiveness in elite handball players. Throwing velocity is undoubtedly a critical factor in the match; the question remains how throwing velocity and accuracy differ under stable and match conditions. Future research should verify how throwing velocity and accuracy in stable conditions affect match performance.
